# Palmitic acid–induced autolysosomal dysfunction and lipotoxicity in neuroinflammation and neurodegeneration

**DOI:** 10.4103/NRR.NRR-D-25-00432

**Published:** 2025-07-05

**Authors:** Eka Norfaishanty Saipuljumri, Jialiu Zeng, Chih Hung Lo

**Affiliations:** 1 Program in Neuroscience & Cognitive Science, University of Arizona, Tucson, AZ, USA; 2 Department of Biomedical and Chemical Engineering, Syracuse University, Syracuse, NY, USA; 3 Interdisciplinary Neuroscience Program, Syracuse University, Syracuse, NY, USA; 4 Department of Biology, Syracuse University, Syracuse, NY, USA

**Keywords:** autolysosomal dysfunction, lipotoxicity, metabolic dysfunction, neuroinflammation, palmitic acid

## Abstract

Neurodegenerative disorders such as Alzheimer’s and Parkinson’s diseases are increasingly associated with metabolic dysfunction, including obesity, type 2 diabetes, and metabolic dysfunction–associated steatotic liver disease. Central to this connection is the dysregulation of lipid metabolism, which extends beyond peripheral tissues to the brain, defective autolysosomal function, oxidative stress, inflammation, and insulin resistance. Lipids, which constitute over half of dry weight of the brain, play critical roles in energy provision, structural integrity, and synaptic function. Dysregulation of lipid metabolism contributes to neuroinflammation, impaired neuronal function, and disrupted blood–brain barrier integrity. Palmitic acid, a saturated fatty acid abundant in high-fat diets, serves as a key model for studying lipid-induced toxicity (lipotoxicity) in the brain. Palmitic acid disrupts autophagy and lysosomal function, mitochondrial function, triggering oxidative stress, contributing to neuroinflammation and neurodegeneration. These effects are particularly pronounced in neurons, which are highly susceptible to lipid-induced toxicity due to their high metabolic demands. Glial cells, including astrocytes, microglia, and oligodendrocytes, also exhibit distinct vulnerabilities and adaptive responses to lipid metabolism dysregulation, further contributing to neuroinflammation and demyelination. Therapeutic strategies, such as supplementation with polyunsaturated fatty acids, AMP-activated protein kinase activation, and lysosome-targeted interventions, show promise in mitigating palmitic acid–induced lipotoxicity and restoring cellular homeostasis. This review comprehensively examines palmitic acid–induced lipotoxicity and its impact on autolysosomal dysfunction across various central nervous system cell types, including neurons, astrocytes, microglia, and oligodendrocytes. Additionally, it highlights therapeutic approaches to restore autolysosomal function under lipotoxic conditions. Advances in multi-omics technologies and a deeper understanding of intercellular crosstalk offer new avenues for developing targeted therapies to restore autolysosomal function, and attenuate neuroinflammation and neurodegeneration.

## Introduction

Literature data have been increasingly associating neurodegenerative diseases, such as Alzheimer’s disease and Parkinson’s disease with metabolic dysfunction. Metabolic disturbances seen in obesity, type 2 diabetes, and metabolic dysfunction-associated steatotic liver disease have emerged as major risk factors for the onset and progression of neurodegenerative diseases (Procaccini et al., 2016; Amorim et al., 2022). Excessive dietary intake and the accumulation of fat deposits in peripheral tissues results in the dysregulation of lipid metabolism and metabolic alterations that are not only confined to the body but also extended to the brain (Sui and Pasco, 2020; Asimakidou et al., 2025; Saeed et al., 2025; Zeng et al., 2025a; Zeng and Lo, 2025). This metabolic imbalance triggers a range of dysfunctions, including inflammation, oxidative stress, defective autophagy, and lysosomal processes, disruption of cellular bioenergetics, and the development of insulin resistance, all of which contribute to brain pathology (Butterfield and Halliwell, 2019; Mizunoe et al., 2019; Vinuesa et al., 2021). The intricate interplay between metabolic disorders and neurodegeneration highlights the critical need to understand how disruptions in lipid homeostasis and metabolic balances contribute to brain disorders (Neto et al., 2023).

Lipids play essential roles in the brain, including an important energy source for the cell, providing structural support, cell signaling, and synaptic transmission, among others (Bazinet and Layé, 2014; Tracey et al., 2021; Zhang et al., 2025). Lipids constitute more than half of the dry weight of the brain, highlighting their crucial role in maintaining brain health (Fantini and Yahi, 2015). Advances in brain lipidomics have revealed strong associations between lipid metabolism and neurodegenerative diseases, underscoring the importance of lipid homeostasis regulation (Osetrova et al., 2024; Ahrends et al., 2025; He et al., 2025; Yoon et al., 2025). Lipidome dyshomeostasis in the brain results in neuroinflammation, impaired neuronal function, and synaptic plasticity, and disrupted blood–brain barrier (BBB) (Custers et al., 2022; Tong et al., 2024). Major lipid classes that make up brain tissue include phospholipids (such as phosphatidylcholines and phosphatidylethanolamines), sphingolipids (primarily ceramides), and sterols (mainly cholesterol) (Osetrova et al., 2024). The composition of these lipids varies across different brain structures. For instance, myelin membranes in oligodendrocytes are particularly rich in sphingolipids, ceramides, and cholesterol, while astrocytes contain neutral lipid deposits (Osetrova et al., 2024). Moreover, 35% of human brain lipids contain omega-3 polyunsaturated fatty acids (PUFAs), with docosahexaenoic acid (DHA) accounting for more than 40% of total omega-3 PUFAs in neuronal tissue, especially in the grey matter (Dighriri et al., 2022). Studies in mice and rat pups have shown that most of the lipids in the brain are produced *de novo* (Edmond et al., 1998; Smith et al., 2024). A similar instance is found in human infant brains, where the majority of the lipids are produced *de novo*, and they are mostly long-chain w-3 fatty acids that support brain cellularity (Björkhem and Meaney, 2004). Through adulthood, *de novo* lipid production such as cholesterol reduces due to multiple reasons, including the declining need for myelin formation in the adult state (Dietschy and Turley, 2004). Fatty acids can also enter the brain via circulation, such as triglycerides transported by apolipoprotein (e.g., apolipoprotein E) or DHA through members of the major facilitator superfamily (Mfsd2a) (Spector, 2001; Bruce et al., 2017; Pifferi et al., 2021).

A widely used model for studying brain lipid metabolism dysregulation involves chronic exposure to high concentrations of palmitic acid (PA), a long-chain saturated fatty acid, which mimics the lipid imbalances seen in obesity and related metabolic disorders (Obaseki et al., 2024). PA treatment of brain cells is an essential tool for understanding the disruptions in cellular homeostasis under lipotoxicity state in the brain (Carta et al., 2017; Ortiz-Rodriguez et al., 2019; Urso and Zhou, 2021; Vesga-Jiménez et al., 2022). PA is the most abundant saturated fatty acid (SFA) found in the circulation (Quehenberger et al., 2010), cerebrospinal fluid (Pilitsis et al., 2001) and the brain (Smith et al., 2024). A recent analysis of adult postmortem brains identified SFAs were the most abundant fatty acid species in the brain (41 ± 0.7 mol%), with PA representing nearly half of total brain saturates and 19 ± 0.7 mol% of total fatty acids (Lacombe et al., 2023). Increased brain uptake and accumulation of PA have been observed in high-fat diet (HFD) fed mice (Vagena et al., 2019; Lim et al., 2021) and in individuals with obesity (Karmi et al., 2010). The brain can also be supplied with PA via direct administration, providing a more direct model suitable for studying lipid metabolic dysregulation in the central nervous system (CNS) (Benoit et al., 2009; Melo et al., 2020).

PA has been shown to prevent autophagy initiation, impair lysosomal acidification, and block autophagosome-lysosome fusion, leading to the accumulation of bioactive lipid intermediates and subsequent lipotoxicity (Hernández-Cáceres et al., 2019, 2020; Melo et al., 2020). Additionally, it can lead to increased neuroinflammation, impaired synaptic plasticity, and neuronal survival (Hernández-Cáceres et al., 2019, 2020; Melo et al., 2020). Restoration of autophagic and lysosomal function has been demonstrated to restore cellular proteostasis and mitochondrial function in brain cells under PA-induced lipotoxicity (Kim et al., 2011; Montero et al., 2020; He et al., 2022). Different CNS cell types exhibit distinct vulnerabilities and adaptive responses to lipid metabolism dysregulation, with neurons being particularly susceptible to lipid-induced toxicity due to their high metabolic demands and limited regenerative capacity (Tracey et al., 2018; Loving and Bruce, 2020; Montani, 2021). Astrocytes, conversely, play a dual role, acting as lipid reservoirs to buffer excess free fatty acids while also contributing to neuroinflammation when overloaded with lipids. Microglia respond by shifting to a pro-inflammatory state, releasing cytokines that exacerbate neurotoxicity and neuronal damage. Oligodendrocytes, which are crucial for maintaining myelin, are also impacted by lipid imbalances, leading to demyelination and impaired neuronal function. There is also intercellular crosstalk between these CNS cell types, influencing lipid metabolism and inflammatory responses in a dynamic manner (Peferoen et al., 2014; de Waard and Bugiani, 2020; Yin et al., 2021; Lan et al., 2024).

**Figure 1:**
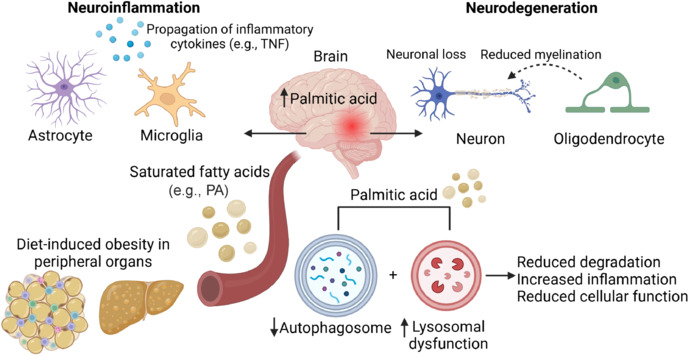
Summary of PA–induced autolysosomal dysfunction, neuroinflammation, and neurodegeneration. Excess of PA accumulated in the blood, cerebrospinal fluid, and brain, contributes to lipotoxicity, thus disrupting the function of key brain cells–neurons, microglia, astrocytes, and oligodendrocytes and promoting neuroinflammation and cell death. The critical mechanisms responsible for these effects are PA–induced erroneous autophagy initiation and diminished lysosomal function, which impair autophagic degradation, thus exacerbating inflammation and compromising cellular homeostasis. Created with BioRender.com. PA: Palmitic acid; TNF: tumor necrosis factor.

In the review presented herein, we provide a comprehensive overview of PA-induced lipotoxicity and its effects on autolysosomal dysfunction across various cell types in the brain (**Figure 1**). We will delve into how fatty acid–induced lipotoxicity disrupts key processes in the autophagy-lysosomal pathway and mitochondrial function, which are critical mechanisms for maintaining cellular homeostasis. Additionally, we examine how these disruptions contribute to neuroinflammation, neuronal death, and the progression of neurodegenerative diseases. Furthermore, we highlight emerging therapeutic strategies aimed at restoring autolysosomal function, attenuating neuroinflammation, and enhancing cell viability under conditions of fatty acid-induced lipotoxicity.

## Search Strategy

Studies cited in this narrative review published from 2001 to 2025 were searched on the PubMed database using the following keywords: palmitic acid, autophagy, lysosomal acidification, lipotoxicity, metabolic dysfunction, obesity, neuroinflammation, and neurodegeneration.

## Lipotoxicity in Neurons

In N43/5 hypothalamic cells, a model of pro-opiomelanocortin (POMC) neurons, exposed to excess cholesterol or PA, there is inhibition of autophagic flux (Barbero-Camps et al., 2018; Hernández-Cáceres et al., 2019; Lee et al., 2020; Roca-Agujetas et al., 2021; He et al., 2022; Levy et al., 2022). PA exposure to N43/5 cells inhibits autophagic flux, further reducing insulin sensitivity (Hernández-Cáceres et al., 2019). PA treatment increased autophagosome puncta, but this effect was not enhanced by BafA1, a lysosomal V-ATPase inhibitor, indicating that lysosomal acidification was already compromised under PA treatment (Hernández-Cáceres et al., 2019). Pharmacological inhibition of free fatty acid receptor 1, for which PA is a ligand, alleviated PA-induced autophagy suppression and improved insulin sensitivity in these neurons (Hernández-Cáceres et al., 2019). In a separate study using N43/5 cells, PA also inhibited autophagic flux (Hernández-Cáceres et al., 2020). However, unlike the first study, PA did not alter lysosomal pH (Hernández-Cáceres et al., 2020). Instead, the authors attributed the reduced autophagic flux to impaired autophagosome–lysosome fusion, which also resulted in significantly enlarged lysosomes, a phenotype commonly linked to disrupted autophagosome–lysosome fusion (Hernández-Cáceres et al., 2020). While both studies report impaired autophagic flux under PA treatment, PA can disrupt autophagy through different mechanisms, including, but not limited to, lysosomal acidification. These findings suggest that the effects of PA on lysosomal acidification may vary and warrant further investigation. In mHypoE-46 hypothalamic neurons, PA exposure impaired autophagic flux and induced endoplasmic reticulum (ER) stress and inflammation, as evidenced by elevated mRNA levels of *Npy*, *Grp78*, and *Il-6* (He et al., 2022). Additionally, PA or stearic acid treatment in POMC neurons reduced total and lysosome-associated membrane protein type 2A, impairing lysosomal function and inhibiting chaperone-mediated autophagy, a selective form of autophagy that degrades specific proteins marked by a KFERQ-like motif via chaperone-mediated lysosomal transport. Inhibition of chaperone-mediated autophagy consequently reduced insulin signaling in POMC neurons (Espinosa et al., 2022). Neuronal exposure to PA in nerve growth factor-differentiated pheochromocytoma cells (NGFDPC12), which adopt a neuronal phenotype upon NGF treatment, induced lysosomal membrane permeabilization (LMP), resulting in the release of cathepsins B, D, and L. This was followed by mitochondrial membrane permeabilization, reactive oxygen species generation, and apoptosis. The authors suggested that PA exposure increases Fas receptor, a death receptor that leads to apoptosis, activates caspase 8, enhances formation of ceramide/sphingosine, and increases BNIP3 and Bax expression, which are biomolecules of the mitochondrial-death signaling pathway, thereby leading to LMP, and lead to the release of lysosomal enzymes (Almaguel et al., 2010). Notably, inhibition of cathepsin L, but not cathepsins B or D, effectively prevented LMP, mitochondrial membrane permeabilization, and apoptosis, highlighting a specific role for cathepsin L in lipid-induced neuronal cell death (Almaguel et al., 2010). The role of cathepsin L in obesity has been explored in both mice and *C. elegans* models (Yang et al., 2007; Naour et al., 2010; Lin et al., 2019). Circulating levels of cathepsin L are significantly elevated in obese mice (Yang et al., 2007), while weight loss in both obese humans and mice reduces these levels (Naour et al., 2010). Moreover, cathepsin L knockout in *C.* *elegans* promoted fat loss (Lin et al., 2019). PA in neuro2a cells significantly decreased the intracellular adenosine triphosphate (ATP) production and increased the levels of mitochondrial superoxide, which could potentially affect lysosomal acidification as shown in other models of PA-induced lipotoxicity (Las et al., 2011), although not studied in (Almaguel et al., 2010). Another study also showed that PA-induced cell death in neurons and astrocytes due to increasing mitochondrial dysfunction and hence increased oxidative stress (Ng and Say, 2018). In lipid-peroxidation product (hydroxynonenal) treated POMC neurons, there are decreased number of lysosomes and an increase number of autophagosomes indicating an inhibition of autophagic flux. In addition, there is increased LMP as indicated by increased levels of cathepsin B and LAMP2 (Levy et al., 2022).

Monounsaturated fatty acids such as oleic acid/oleate (OA) and PUFAs have been shown to mitigate the toxic effects of PA-induced lipotoxicity. Palmitate induces neuroinflammation and ER stress in hypothalamic mHypoA-POMC neurons through activating the extracellular signal-regulated kinase and Jun N-terminal kinase pathway that increases inflammation and are prevented by treatment with oleate (Tse and Belsham, 2018). Co-incubation with oleate restores PA-mediated impairment of autophagy and prevents cell death cells (He et al., 2022). OA reduced LC3B and p62 expression levels in N43 cells, suggesting enhanced autophagic clearance, and reduced inflammatory cytokines interleukin-6 (IL-6) and tumor necrosis factor (TNF) levels (He et al., 2022). In PA-treated NGFDPC12 cells, DHA, a PUFA, increased mRNA levels of autophagy proteins LC3-II, ATG7, and ATG12, while reducing caspase activity and reactive oxygen species levels, thereby improving cell viability (Montero et al., 2020). Other PUFAs such as eicosapentaenoic acid, arachidonic acid, n-3 docosapentaenoic acid, and n-6 docosapentaenoic acid similarly enhanced neuronal cell survival and reduced inflammation following PA exposure (Rodriguez-Navas et al., 2016; Montero et al., 2020). Pre-treatment with OA in neuro-2a cells prior to PA exposure stimulated ATP production in mitochondria and reduced PA-induced cytotoxicity (Kwon et al., 2014). Activation of AMP-activated protein kinase (AMPK) pathway, a positive regulator of autophagy, with 5-aminoimidazole-4-carboxamide ribonucleotide and thienopyridine inhibits apoptosis and tau hyperphosphorylation mediated by PA in SH-SY5Y cells (Kim et al., 2011).

HFDs are rich in cholesterol and various SFAs, including PA (Librán-Pérez et al., 2015). HFD mice on 6 weeks of diet showed increased PA and stearic acid deposition in the hippocampus, which led to increased insulin resistance, and overexpression of the palmitoyltransferase zDHHC3, which leads to impaired synaptic plasticity (Spinelli et al., 2017). In another study, 7 days of HFD resulted in increased levels of PA in mice brains as indicated by fatty acid composition analysis. These levels continued to increase until 56 days (Gimenez da Silva-Santi et al., 2018). When compared to mice fed on a high carbohydrate diet, it was shown that HFD mice had a faster brain deposition of fatty acid (FA), reaching the maximum FA accumulation on day 14 whereas high carbohydrate diet reached maximum FA deposition on day 14 or 28. This could potentially indicate increased amounts of PA circulation to the brain. Additionally, there is increased expression of inflammatory cytokines such as TNF and IL-6 due to PA accumulation in the brain (Gimenez da Silva-Santi et al., 2018). Eight weeks of HFD feeding led to decreased autophagy and brain-derived neurotrophic factor levels, activated microglia and astrocytes, increased neuroinflammation, and depressive and anxiety-like behaviors in mice. HFD feeding inhibits AMPK phosphorylation and increases mammalian target of rapamycin (mTOR) phosphorylation, leading to an inhibition of autophagy. Treatment with mTOR inhibitor rapamycin increased autophagy and brain-derived neurotrophic factor levels, and reduced neuroinflammation (Li et al., 2022). In wild-type and 5×FAD transgenic mice, a mouse model which recapitulates key AD phenotypes with robust amyloid pathology, 16 weeks of HFD feeding leads to memory deficits and impaired proteolytic function, resulting in increased amyloid-beta accumulation (Sarroca et al., 2021). Resveratrol reduced the amyloid burden aggravated by HFD in 5×FAD and protected against HFD-induced tau pathology in both wild-type and 5×FAD strains, as well as prevented memory loss in both wild-type mice under HFD and in 5×FAD mice. Mechanistically, resveratrol restored the proteolytic activity, increased AMPK protein levels indicative of increased autophagy function, and upregulated sirtuin 1 pathway, which plays a role in increasing mitochondrial biogenesis and attenuating oxidative stress (Sarroca et al., 2021). Using an AD mouse model (APP-PSEN1-SREBF2), it was shown that elevated brain cholesterol levels promoted autophagosome formation but impaired their fusion with endosomal–lysosomal vesicles. The disruption in the autophagy process hindered the degradation of amyloid-beta and endogenous tau (Barbero-Camps et al., 2018). In a follow-up study using cholesterol-enriched SH-SY5Y cells and cultured primary neurons, elevated cholesterol levels induced mitochondrial accumulation of PINK1, a kinase that senses mitochondrial damage and initiates quality control through mitophagosome formation, while simultaneously impairing lysosomal-mediated clearance of these mitophagosomes. Moreover, in aged mice, chronic cholesterol accumulation results in an age-dependent impairment of autophagy receptor optineurin translocation to mitochondria, thereby impairing autophagic clearance of damaged mitochondria, leading to increased oxidative stress (Roca-Agujetas et al., 2021).

To investigate the direct effects of PA on brain changes, PA was administered directly to the mice in other studies. A single PA treatment was given via intraperitoneal injection to Swiss mice for 2 and 24 hours. Mice exposed to PA for 24 hours exhibited anxiety-like behavior without impairment in locomotion, food intake, depressive-like behavior, or spatial memory (Moon et al., 2014). This was accompanied by disrupted dopamine metabolism, which is responsible for the anxiety behavior, however further investigation into the defects of serotonergic signaling is needed to identify PA mechanistic implication in dopamine metabolism (Moon et al., 2014). In another study, intracerebroventricular infusion of palmitate at 22 or 66 μg/mL cerebrospinal fluid for 10 days increases inflammatory TNF levels, induces astrocyte and microglial activation in the mouse hippocampus, and impairs synaptic plasticity and memory (Melo et al., 2020). One thing to note is that in this study, the concentrations used are 3.5 to 10-fold higher than what was found in the cerebrospinal fluid of obese individuals (Melo et al., 2020). A similar study administering PA at 180 μmol/L for 8 days via intracerebroventricular route resulted in changes in autophagy-related gene profiles (Reginato et al., 2020). However, the PA effect on autophagic flux was not assessed in this study (Reginato et al., 2020).

These studies underscore how PA disrupts autophagy and lysosomal function in hypothalamic and neuronal cells through impaired acidification, autophagosome–lysosome fusion, and lysosomal membrane integrity. These defects drive insulin resistance, oxidative stress, inflammation, and cell death, all hallmarks of obesity-related neurodegeneration. Promising interventions, including AMPK activators, unsaturated fatty acids, and autophagy enhancers, can reduce PA-induced damage. Still, more *in vivo* studies are needed to clarify the mechanisms and refine therapies that restore lysosomal function and protect neurons from lipotoxicity.

## Glial Activation and Loss of Functionality

Glial cells, including oligodendrocytes, microglia, and astrocytes utilize lipids for distinct functions. Oligodendrocytes are enriched in sulfatide and hexosylceramide to support myelination processes (Eckhardt, 2008; Montani, 2021). Microglia play a critical role in myelination and remyelination and are enriched in sphingomyelin, a lipid largely absent in neurons (Schneider et al., 2019; Chausse et al., 2021; Alashmali, 2024). Astrocytes, which maintain the BBB, modulate synaptic circuits, and recycle neurotransmitters, exhibit higher levels of diacylglycerol, which is essential for lipid metabolism (Kolczynska et al., 2020; Lee et al., 2021). Excessive dietary SFAs have been shown to impair autolysosomal and mitochondrial functions, triggering inflammatory responses and impairing normal cellular processes.

Astrocytes are the most abundant glial cells in the CNS and regulate lipid metabolism by processing fatty acids, cholesterol, phospholipids, and sphingolipids (Zhang et al., 2024). Primary astrocytes from mouse cerebral cortex treated with PA showed increased LC3-II and p62 expression, with immunofluorescence revealing cytoplasmic p62 puncta, indicative of impaired autophagic flux (Ortiz-Rodriguez et al., 2019). Interestingly, when hydroxychloroquine, an inhibitor of lysosomal acidification, was co-treated with PA, there was no further elevation of LC3-II levels, indicating that PA already has impaired lysosomal acidification (Ortiz-Rodriguez et al., 2019). In another study using primary culture of microglia and astrocytes from rats, treatment with PA triggered the release of inflammatory cytokines TNF and IL-6 from astrocytes. Depletion of microglia from primary astrocyte cultures using the lysosomotropic agent L-Leucine methyl ester revealed that the ability of PA to trigger cytokine release is not dependent on the presence of microglia (Gupta et al., 2012). In cultured astrocytes treated with PA or stearic acid, there is reduced mitochondrial membrane potential and diminished respiratory chain enzyme activity, indicative of impaired mitochondrial function (Schmitt et al., 2024). In PA treated hippocampal astrocytes, there is increased ER stress leading to cell death in astrocytes. Treatment with estradiol increased the levels of anti-inflammatory cytokine IL-10, along with decreased phosphorylated Jun N-terminal kinase and TNF levels, indicative of reduced inflammation, along with reduced caspase-3 activation indicative of reduced apoptosis (Frago et al., 2017). In mice with 1 or 3 days of HFD feeding, there is increased hypothalamic inflammatory signaling as shown by higher TNF levels, and the presence of reactive gliosis in the hypothalamic arcuate nucleus (Thaler et al., 2012).

In PA-treated BV-2 microglial cells, autophagy proteins ATG7, ATG5, and LC3I/II levels were reduced, while p62 levels increased, indicating reduced autophagosome formation and impaired autophagic degradation, suggesting reduced lysosomal degradative function (Cui et al., 2021). Additionally, there is increased inflammatory response, upregulation of TNF, IL-1β, and IL-6 levels, and reduced BV-2 cell viability (Cui et al., 2021). Melatonin treatment increases autophagy function through downregulating the toll-like receptors 4 (TLR4)/phosphorylated-protein kinase B (Akt)/mTOR signaling pathway, by decreasing the levels of TLR4, Akt, and phosphorylated-mTOR, leading to increased LC3-II expression and reduced cell apoptosis (Cui et al., 2021). Proteomic analysis of extracellular vehicles (EVs) secreted by PA-treated BV-2 cells showed increased levels of lysosomal V-ATPase subunit ATP6AP1, suggestive of impairment of lysosomal function (De Paula et al., 2024). Additionally, it has been shown in other studies that EV release can be a result of autolysosomal dysfunction (Eitan et al., 2016; Miranda et al., 2018). When the EVs secreted from PA-exposed microglia are administered intra-cerebroventricularly to mice, the mice had memory impairment, depression-like behavior, and glucose intolerance (De Paula et al., 2024). In high-carbohydrate, high-fat diet-fed mice and PA-treated BV-2 cells, mitochondrial structural abnormalities are observed, as visualized by disrupted cristae and reduced matrix electron density (Gao et al., 2017). PA exposure also led to reduced mitochondrial function as shown by reduced mitochondrial respiration and membrane potential in BV-2 cells (Hidalgo-Lanussa et al., 2018; De Paula et al., 2024), which further lead to increased reactive oxygen species production, and decreased levels of anti-apoptotic protein neuroglobin, thereby contributing to elevated cell death (Hidalgo-Lanussa et al., 2018). Tibolone treatment enhanced antioxidant defense by upregulating superoxide dismutase and catalase, preserving mitochondrial function and improving cell viability (Hidalgo-Lanussa et al., 2018). Additionally, pre-treating BV-2 cells with linoleic acid before PA exposure mitigated apoptosis and suppressed IL-6 expression through inhibiting extracellular signal-regulated kinase phosphorylation (Tu et al., 2019).

Excessive lipid accumulation in oligodendrocytes disrupts their metabolic stability, impairing myelination and viability. PA and high glucose treatment induced insulin resistance in human oligodendrocytes. Additionally, the effect was propagated to recipient neurons when EVs from PA-treated oligodendrocytes treated recipient neurons increased neuronal insulin resistance (Kim et al., 2023). Mice with HFD feeding for 12 weeks showed loss of oligodendrocyte progenitors across the brain and spinal cord. Transcriptomic and metabolomic analysis showed an increase in ER stress, mitochondrial dysfunction, and oxidative stress in these mice (Langley et al., 2020). In primary oligodendrocytes (O-2A) and their oligodendrocyte progenitor cells, treatment with psychosine (galactosylsphingosine) led to lysosomal alkalization (Folts et al., 2016). In 158N oligodendrocytes treated with very-long-chain fatty acids or 7-ketocholesterol, there is increased autophagosome and p62 accumulation, indicative of impaired autophagic flux (Nury et al., 2018; Doria et al., 2019). Transmission electron microscopy analysis further revealed lysosome mislocalization, aggregation, and destabilization, indicative of impairment of lysosomal function, which is further confirmed by confocal microscopy imaging with lysosome-specific dyes (Doria et al., 2019). Additionally, 7-ketocholesterol treatment suppressed peroxisome proliferator–activated receptor expression, leading to increased mitochondrial dysfunction in oligodendrocytes (Badreddine et al., 2017; Doria et al., 2019). Prolonged exposure to very-long-chain fatty acids, 7-ketocholesterol, or psychosine also triggered oligodendrocyte cell death (Folts et al., 2016; Badreddine et al., 2017; Doria et al., 2019). Treatment with therapeutic agents such as chlorotrianisene, colforsin, clofoctol, tulobuterol, or insulin-like growth factor 1 to oligodendrocytes can potentially rescue lysosomal acidification defects via increasing the levels of cyclic adenosine monophosphate (Folts et al., 2016).

These findings highlight the distinct roles of oligodendrocytes, microglia, and astrocytes in lipid metabolism and how each is uniquely affected by excess dietary lipids such as PA. PA impairs autophagy, mitochondrial function, and lysosomal integrity across glial types, driving inflammation, cell death, and dysfunction in key brain processes such as myelination, synaptic plasticity, and glucose regulation. Targeting autophagic pathways and mitochondrial health may help counteract PA-induced damage. Further research is needed to clarify the mechanisms of lipid-driven glial dysfunction and identify strategies to restore cellular balance in neurodegenerative disease.

## Broader Implications and Future Perspectives

**Figure 2:**
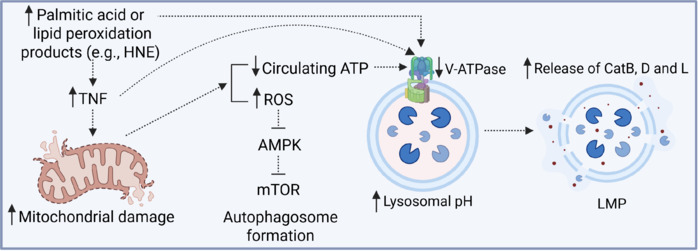
Converging pathways on how lipotoxicity affects autolysosomal and mitochondrial function in neuron and glial cells. Saturated fatty acids or lipid peroxidation products such as HNE induce the elevation of inflammatory cytokines such as TNF, which further induce mitochondrial damage. Mitochondria damage results in the release of ROS, and reduced ATP for lysosomal V-ATPase use, therefore reducing lysosomal acidification. ROS production can also inhibit AMPK levels, which results in the activation of mTOR and hence inhibition of autophagy or autophagosome formation. Lysosomes with increased pH or reduced acidification can result in LMP, which leads to cytosolic release of lysosomal cathepsins B, D, and L (CatB, D, and L), which can propagate further inflammation and cellular death. Created with BioRender.com. AMPK: AMP-activated protein kinase; ATP: adenosine triphosphate; Cat: cathepsin; HNE: 4-hydroxynonenal; LMP: lysosomal membrane permeabilization; mTOR: mammalian target of rapamycin; ROS: reactive oxygen species; V-ATPase: vacuolar-type adenosine triphosphatase.

Accumulating evidence has shown that chronic exposure to PA and other lipid peroxidation products in neuronal and glial cells impairs autolysosomal function, and mitochondrial function, leading to increased inflammation and cell death (**Figure 2**). HFD feeding in mice exacerbates these effects, contributing to cognitive decline, neuroinflammation, and synaptic impairments in mouse models of neurodegeneration. Neuroprotective therapeutic interventions, such as OA, DHA, resveratrol, and AMPK activation, mitigate PA-induced lipotoxicity by enhancing autophagic function, reducing inflammation, and restoring mitochondrial function. Despite these insights, several key questions remain. In particular, the mechanisms by which PA disrupts lysosomal acidification and autophagosome–lysosome fusion in brain cells are still poorly understood. In cardiomyocytes, PA has been shown to impair lysosomal function by inducing dissociation of V-ATPase subunits, a proton pump critical for maintaining lysosomal acidity (Liu et al., 2017). Whether similar disruptions occur in neurons or glia remains to be determined. Further investigation is needed to clarify how PA affects lysosomal V-ATPase assembly and/or lysosomal membrane stability in the brain.

While most lipid accumulation in the body stems from dietary intake, genetic factors also play a significant role in regulating lipid metabolism and distribution. Triggering receptor expressed on myeloid cells 2 (TREM2) is a single-pass transmembrane immune receptor selectively expressed in microglia within the CNS. TREM2 senses lipids and mediates myelin phagocytosis. A study has shown that TREM2 is a key transcriptional regulator of cholesterol transport and metabolism under conditions of chronic myelin phagocytic activity, as TREM2-deficient microglia fail to clear myelin cholesterol, resulting in cholesteryl ester (pathogenic lipids) accumulation (Nugent et al., 2020). *APOE4* genetic mutation leads to impaired cholesterol transport, leading to aberrant lipid accumulation (Yang et al., 2023).

**Figure 3:**
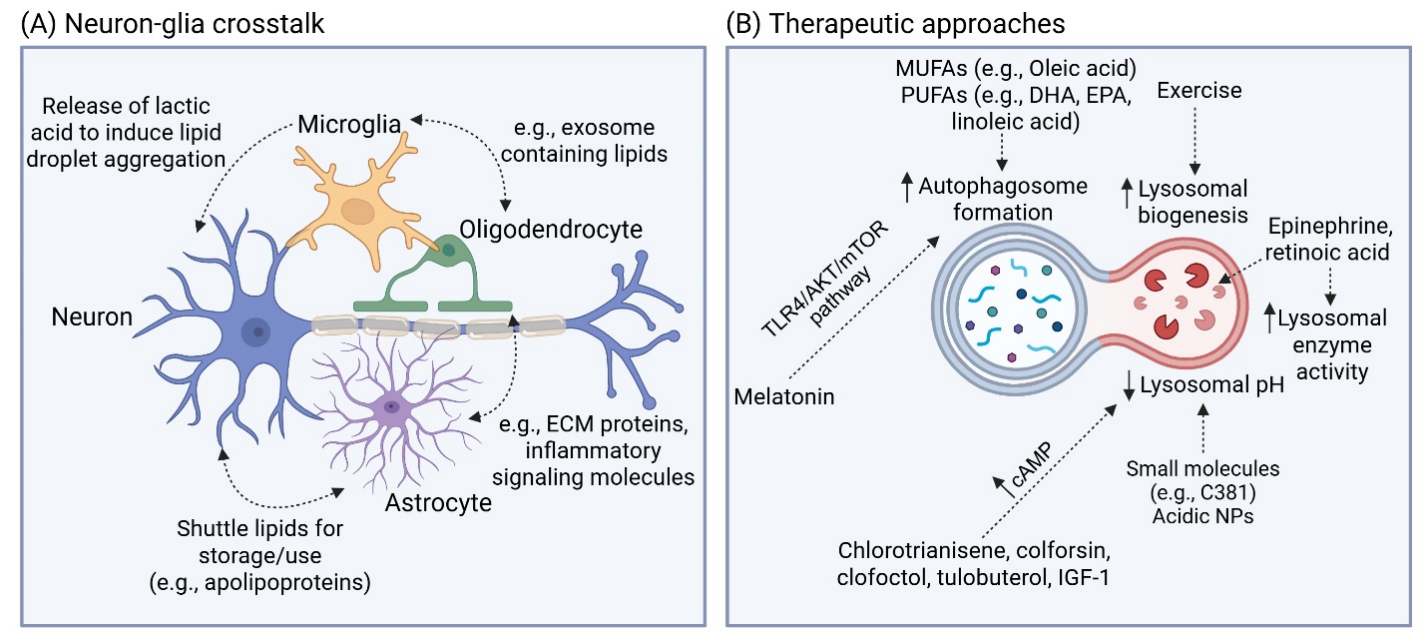
Neuron-glia crosstalk and therapeutic strategies to improve autolysosomal function in neuron and glial cells. (A) Different central nervous system cells can crosstalk with each other under lipotoxicity or inflammatory states. Astrocytes and neurons can shuttle lipoproteins between each other. Microglia under inflammation can release lactic acid which can induce lipid droplets formation in neurons. Microglia-oligodendrocyte interaction can occur via exosome transfer. Astrocyte-oligodendrocyte interaction can be mediated by ECM proteins and inflammatory signaling molecules. (B) Summary of different therapeutic agents that can either modulate cellular signaling pathways to increase autophagosome formation or increase lysosomal acidification or lysosomal enzyme activity. Melatonin promotes autophagosome formation by modulating the TLR4/AKT/mTOR signaling pathway. MUFAs (e.g., oleic acid) and PUFAs (e.g., DHA, EPA, linoleic acid) also stimulate autophagy. Physical exercise enhances lysosomal biogenesis. Several small molecules including chlorotrianisene, colforsin, clofoctol, tulobuterol, and IGF-1 lower lysosomal pH via increasing cAMP levels. Additionally, compounds such as C381 and acidic NPs have been shown to restore lysosomal acidification. Epinephrine and retinoic acid are known to enhance lysosomal enzyme activity. Collectively, strategies that simultaneously promote autophagy and lysosomal function synergistically improve autolysosomal fusion and cargo degradation. Created with BioRender.com. AKT: Protein kinase B; cAMP: cyclic adenosine monophosphate; DHA: docosahexaenoic acid; ECM: extracellular matrix; EPA: eicosapentaenoic acid; IGF-1: insulin-like growth factor 1; mTOR: mammalian target of rapamycin; MUFAs: monounsaturated fatty acids; NP: nanoparticle; PUFAs: polyunsaturated fatty acids; TLR: Toll-light receptor.

Brain lipid metabolism varies by region, cell compositions, and energy demands (Bruce et al., 2017; Hofmann et al., 2017). For instance, the hypothalamus contains more FFAs and triglycerides to regulate energy balance and feeding behavior, whereas the frontal cortex is enriched in cholesterol and sphingolipids, essential for membrane stability and neural signaling (Fitzner et al., 2020; Lee et al., 2022; Mota-Martorell et al., 2022). Cortical astrocytes contain cholesterol and other lipids to support the BBB and cell adhesion, while hippocampal astrocytes are rich in sphingolipids, aiding synaptic plasticity in learning and memory (Wang et al., 2022; Zhang et al., 2024). Hence, understanding region- and cell type–specific lipidomics could improve strategies to mitigate lipotoxicity (O’Connor et al., 2023b; Giblin et al., 2025). Furthermore, interactions between neurons and glial cells are crucial for fatty acid metabolism, storage, and survival. Neurons have high energy demands to sustain membrane potential and synaptic transmission, making them heavily dependent on glucose, whereas glial cells exhibit greater metabolic flexibility (Barber and Raben, 2019). Crosstalk between these cells often occurs in response to energy fluctuations or inflammation (**Figure 3A**). For instance, astrocytes and neurons exchange lipids via apolipoproteins to regulate the amount of FFAs, particularly in hyperactive neurons (Ioannou et al., 2019; Yin et al., 2021). Astrocytes are also able to convert excess FFAs into lipid droplets for storage (Ioannou et al., 2019). Conversely, microglia interact with neurons by inducing lipid droplet accumulation in neurons via microglial-derived lactic acids (Lan et al., 2024). Oligodendrocyte-microglia crosstalk has been proposed to be mediated via exosomes, where exosomes isolated from mouse oligodendrocytes are enriched in ceramide and myelin proteins and could propagate to microglia (Peferoen et al., 2014). Between oligodendrocytes and astrocytes, their crosstalk involves extracellular matrix proteins and inflammatory signaling molecules (de Waard and Bugiani, 2020). More research is needed to elucidate precise mechanisms involving more than two interacting cell types under lipotoxicity conditions.

Mitochondria–lysosome crosstalk can play an important role in mediating lipotoxicity. Mitochondria primarily oxidize these fatty acids into acetyl-CoA for energy production in the brain, while lysosomes degrade lipids into smaller fatty acids (Audano et al., 2018). Impairment of autolysosomal function reduces the clearance of toxic lipids and reduces autophagy turnover of dysfunctional mitochondria (Assali et al., 2019). Conversely, lipotoxicity and increased lipid peroxidation products can impair mitochondrial integrity and function (Xiao et al., 2017), which can lead to reduced availability of ATP for essential lysosomal V-ATPase function (Las et al., 2011; Assali et al., 2019). In mouse brains with mitochondrial protein AIF knock-out, the presence of mitochondrial dysfunction resulted in the formation of large, poorly acidified lysosomes, and impairment of lysosomal activity (Demers-Lamarche et al., 2016). Together, these findings highlight a critical, bidirectional relationship between mitochondria and lysosomes in regulating lipid homeostasis, where dysfunction in one organelle exacerbates the failure of the other, amplifying lipotoxic stress and contributing to neurodegeneration.

Restoring autolysosomal dysfunction is important to prevent neuroinflammation and neurodegeneration under lipotoxicity conditions. Multiple studies have explored the use of lysosome-targeted therapeutics, including small molecules such as C381, and nanoparticles that release acidic components to restore lysosomal pH, to restore lysosomal acidification impairment in different neurodegenerative and metabolic disease models (Lo and Zeng, 2023; Quick et al., 2023; Lo, 2024; Lo et al., 2024; Zeng et al., 2025a, b), and could potentially be applied to other neurodegenerative disease models induced by lipotoxicity (**Figure 3B**). Other therapeutic approaches to increase lysosomal function include the activation of mannose-6-phosphate receptors with epinephrine or retinoic acid to increase lysosomal enzyme, β-glucuronidase, and activity (Critchley et al., 2022). Additionally, lysosome-targeted therapeutics could be modified with groups or moieties that enhance their ability to effectively cross the BBB to target brain cells (Asimakidou et al., 2024b). Lifestyle interventions, such as exercise, dietary restriction, and strategies to reduce systemic inflammation, can help restore brain lipidome dyshomeostasis through body-brain interactions (Asimakidou et al., 2024a, 2025). In mouse models, long-term exercise has been shown to enhance lysosomal function by promoting lysosomal biogenesis and enzyme activity (Huang et al., 2019). A combinatorial approach targeting both autolysosomal dysfunction (Udayar et al., 2022; Lo and Zeng, 2023; Quick et al., 2023) and neuroinflammation via anti-inflammatory treatments (Adamu et al., 2024; Zeng et al., 2024; Lo, 2025a, b) may be effective in mitigating lipotoxicity-associated neurodegeneration and warrants further investigation.

Additionally, understanding the interactions between lipids and various species and conformations of intrinsically disordered proteins is important (Luäcke et al., 2006; Deryusheva et al., 2019; Lo and Sachs, 2021; Lo, 2022). Advances in multi-omics technologies offer the potential to dissect the molecular heterogeneity of specific cell types involved in the development of neurodegenerative diseases (Fangma et al., 2023; O’Connor et al., 2023a, b; Woldemariam et al., 2023). Through using these approaches, we can address the complex body-brain interactions and provide a clearer roadmap for developing more effective treatments. This review not only aims to consolidate our understanding of palmitate-induced autolysosomal dysfunction and lipotoxicity but also to shed light on future therapeutic avenues that could hold promise for tackling neurodegenerative diseases in a personalized and targeted manner.

## Data Availability

*Not applicable*.
